# Analysis of Transduction Efficiency, Tropism and Axonal Transport of AAV Serotypes 1, 2, 5, 6, 8 and 9 in the Mouse Brain

**DOI:** 10.1371/journal.pone.0076310

**Published:** 2013-09-27

**Authors:** Dominik F. Aschauer, Sebastian Kreuz, Simon Rumpel

**Affiliations:** 1 Research Institute of Molecular Pathology (IMP), Vienna, Austria; 2 Target Discovery Research, Boehringer Ingelheim Pharma GmbH & Co. KG, Biberach an der Riß, Germany; University of Kansas Medical Center, United States of America

## Abstract

Recombinant Adeno-associated virus vectors (rAAV) are widely used for gene delivery and multiple naturally occurring serotypes have been harnessed to target cells in different tissues and organs including the brain. Here, we provide a detailed and quantitative analysis of the transduction profiles of rAAV vectors based on six of the most commonly used serotypes (AAV1, AAV2, AAV5, AAV6, AAV8, AAV9) that allows systematic comparison and selection of the optimal vector for a specific application. In our studies we observed marked differences among serotypes in the efficiency to transduce three different brain regions namely the striatum, hippocampus and neocortex of the mouse. Despite the fact that the analyzed serotypes have the general ability to transduce all major cell types in the brain (neurons, microglia, astrocytes and oligodendrocytes), the expression level of a reporter gene driven from a ubiquitous promoter varies significantly for specific cell type / serotype combinations. For example, rAAV8 is particularly efficient to drive transgene expression in astrocytes while rAAV9 appears well suited for the transduction of cortical neurons. Interestingly, we demonstrate selective retrograde transport of rAAV5 along axons projecting from the ventral part of the entorhinal cortex to the dentate gyrus. Furthermore, we show that self-complementing rAAV can be used to significantly decrease the time required for the onset of transgene expression in the mouse brain.

## Introduction

Viral vectors allow temporally and spatially controlled expression of genes of interest in various tissues and have therefore become a widely-used tool in the biosciences, including neurobiology [1,2]. In recent years many studies have taken advantage of recombinant viral vectors derived from Adeno-associated virus (AAV) as a gene delivery tool [3-5]. AAV is a single-stranded DNA virus with a small (~20nm) protein capsule that belongs to the family of parvoviridae [6,7]. Due to its inability to replicate in the absence of helpervirus co-infections -typically Adenovirus or Herpesvirus infections- AAV is often referred to as dependovirus. AAV infections produce only mild immune responses and are considered to be nonpathogenic, a fact that is also reflected by lowered biosafety level requirements for the work with recombinant AAVs (rAAV) compared to other popular viral vector systems. Within transduced cells rAAV vector genomes persist as episomal concatemers, thereby limiting the risk of insertional mutagenesis. Due to its low immunogenicity and the absence of cytotoxic responses AAV-based expression systems offer the possibility to express genes of interest for months in quiescent cells [8,9].

Production systems for rAAV vectors typically consist of a DNA-based vector containing a transgene expression cassette, which is flanked by inverted terminal repeats derived from AAV2. Construct sizes are limited to approximately 4.7-5.0 kb, which corresponds to the length of the wild-type AAV genome. rAAVs are produced in cell lines. The expression vector is co-transfected with a helper plasmid that mediates expression of the AAV rep genes which are important for virus replication and cap genes that encode the proteins forming the capsule. In addition, specific Adenovirus genes that are required for replication are expressed in trans [10]. In recent years a plethora of naturally occurring AAV serotypes has been described, which primarily differ in surface properties of the capsid [11-13]. By combining AAV2-based genomic constructs with cap genes derived from different serotypes the generation of so called pseudotyped rAVVs is feasible. Currently packaging systems for about 10 different serotypes are available for construction of vectors [11,14].

Many intermediate steps of the wild-type infection cycle of AAV depend on specific interactions of the capsid proteins with the infected cell. These steps include primary recognition of surface receptors, induction of endocytosis, release from the endosomal compartment, transport to and entry into the nucleus and final unpackaging of the viral DNA from the capsid [15]. These interactions are crucial determinants of efficient transduction and expression of genes of interest when rAAV is used as gene delivery tool. Indeed, significant differences in transduction efficacy of various serotypes for particular tissues and cell types have been described [11,12,16-19]. For efficient expression of genes of interest it is therefore crucial to choose a suitable serotype. To make an informed choice on the most appropriate serotype for a specific application it is important to have quantitative data on the transduction properties of the various serotypes in comparable conditions.

Here, we provide an in-depth resource that allows direct comparisons of various serotypes for gene delivery in the mouse brain, including also serotypes only recently used for gene delivery. We analyzed the properties of AAV 1, 2, 5, 6, 8 and 9 pseudotyped vectors for driving expression of the reporter gene GFP in three different regions of the mouse brain. We choose the hippocampus as this is an intensely studied brain region for its role in memory formation and spatial navigation [20], the striatum for its role in the coordination of movements and neurological diseases such as Parkinson’s disease [21,22] and finally the neocortex as the part of the brain specific to mammals that mediates higher-order brain functions such as perception and memory [23]. Specifically, we focused on the part of the neocortex that plays a major role in the perception of sounds, the auditory cortex. For each serotype we quantified the general transduction efficacy, tropism for the major cell types in the brain, potential to induce an immune reaction and its axonal transport within the nervous system. Furthermore, we demonstrate that self-complementary viruses offer an option to accelerate the expression of genes of interests in neurons *in vivo*.

## Methods

### Molecular cloning of rAAV constructs

**Figure 1:**
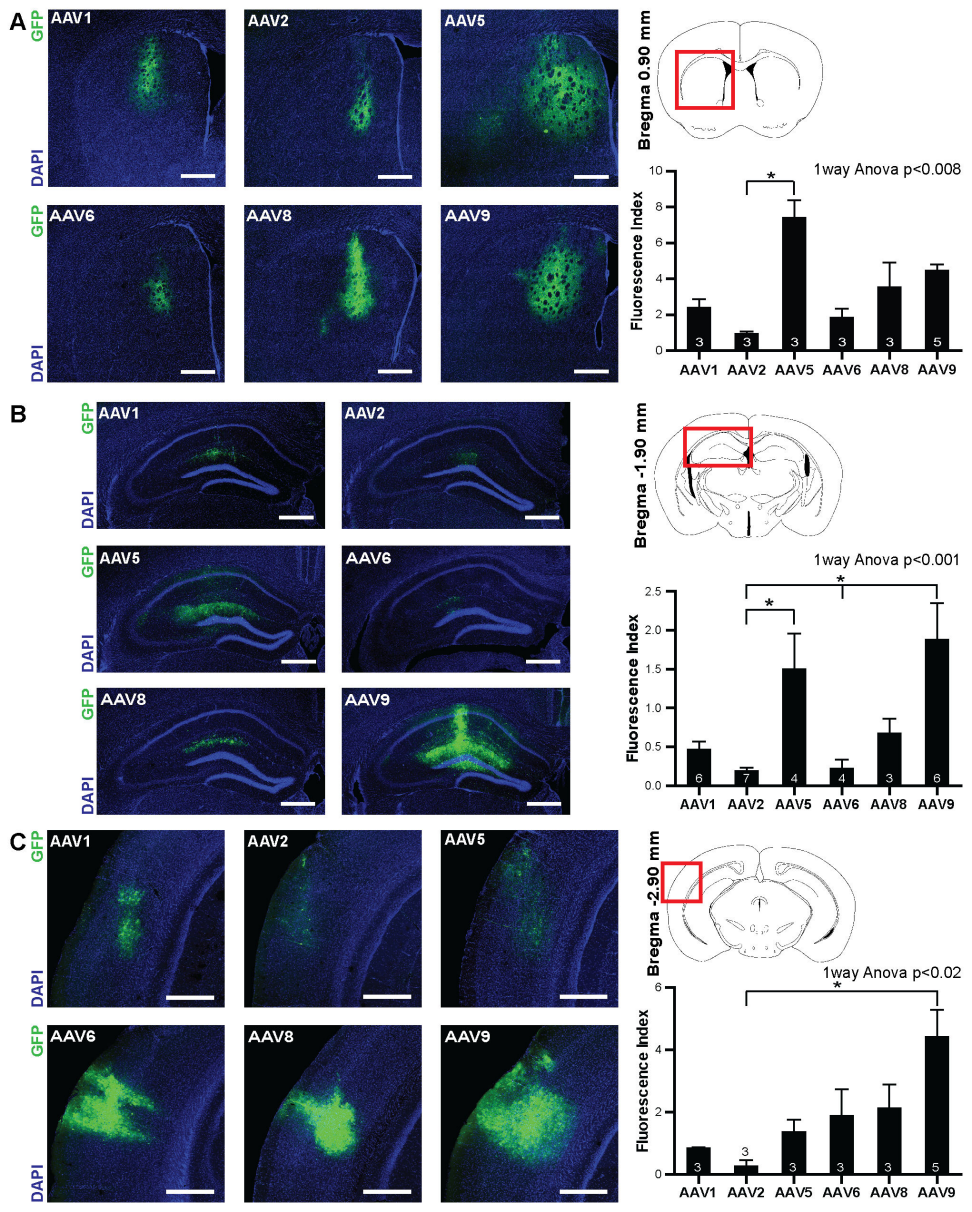
General expression efficacy in striatum, hippocampus and auditory cortex. A) Left: six confocal fluorescence images of the striatum taken from mice injected with various AAV serotypes as indicated in the panels. Blue: DAPI, green: GFP. Right, top: schematic of a coronal brain section with red box indicating the position of the images shown on the left with reference to Bregma. Right, bottom: Mean fluorescence index (see methods) for the striatal sections measured following transduction with the different serotypes. Number of analyzed mice shown at the bottom of the bars. B) Same as A), however, showing images and data from mice that received injections in the hippocampus. C) Same as A), however, showing images and data from mice that received injections in the auditory cortex. Acquisition and display settings for images from a given brain area are the same for different serotypes. All scale bars: 500 µm. All bars represent mean±SEM. Asterisks indicate significant differences at the p<0.05 level.

**Figure 2:**
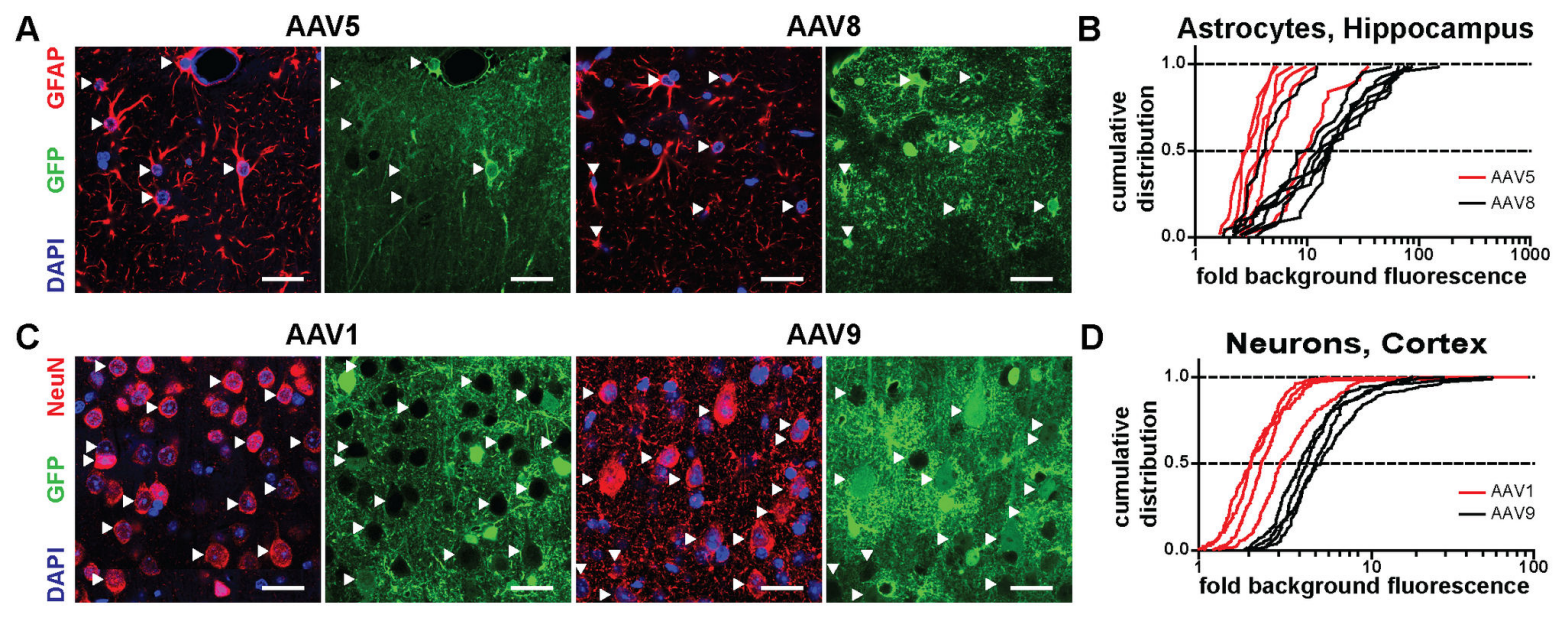
Expression efficacy in immunohistochemically identified cell-types. A) Confocal images of brain sections of the hippocampus prepared from mice that received injections of rAAV5 (left) and rAAV8 (right). Red channel: immunohistochemical label for GFAP, an astrocyte marker; blue channel: DAPI, labeling nuclei, green channel: GFP expression driven by viral vector. Examples of individual immunohistochemically identified cell bodies are marked with white arrowheads. B) Cumulative distributions of fluorescence measurements from individual cells expressed as ‘fold background’ (see methods). Individual lines correspond to a given analyzed section. Note, that cumulative distributions for astrocytes obtained from rAAV8 transduced mice are systematically shifted towards higher fluorescence levels as compared to rAAV5 transduced mice. C) Confocal images of brain sections of the auditory cortex prepared from mice that received injections of rAAV1 (left) and rAAV9 (right). Red channel: immunohistochemical label for NeuN, a neuron marker; blue channel: DAPI, labeling nuclei, green channel: GFP expression driven by viral vector. Examples of individual immunohistochemically identified cell bodies are marked with white arrowheads. D) Cumulative distributions of fluorescence measurements from individual cortical neurons expressed as fold background. All scale bars: 25 µm.

**Figure 3:**
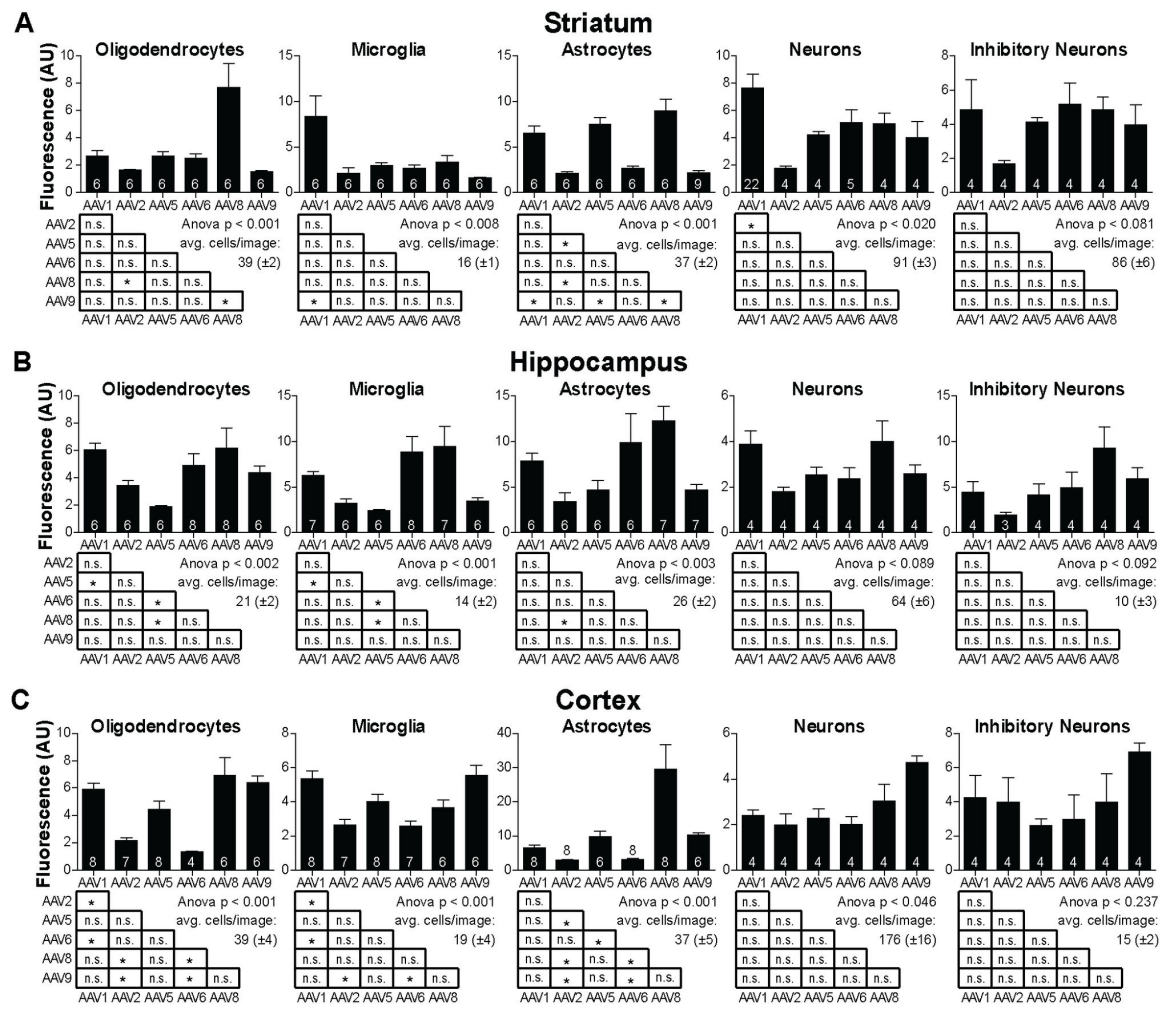
Summary of cell-type specific expression analysis. A) Data obtained from striatum: average of the median florescence levels for oligodendrocytes, microglia, astrocytes, neurons and inhibitory neurons for all of the analyzed six serotypes. Number of analyzed sections is shown at the bottom of the bars. Statistical analysis of fluorescence measurements shown below bar graphs. Asterisks indicate significant differences at the p<0.05 level. B) same as A), however, showing data obtained from hippocampal injections. C) same as A), however, showing data obtained from auditory cortex injections. All bars represent mean±SEM.

**Figure 4:**
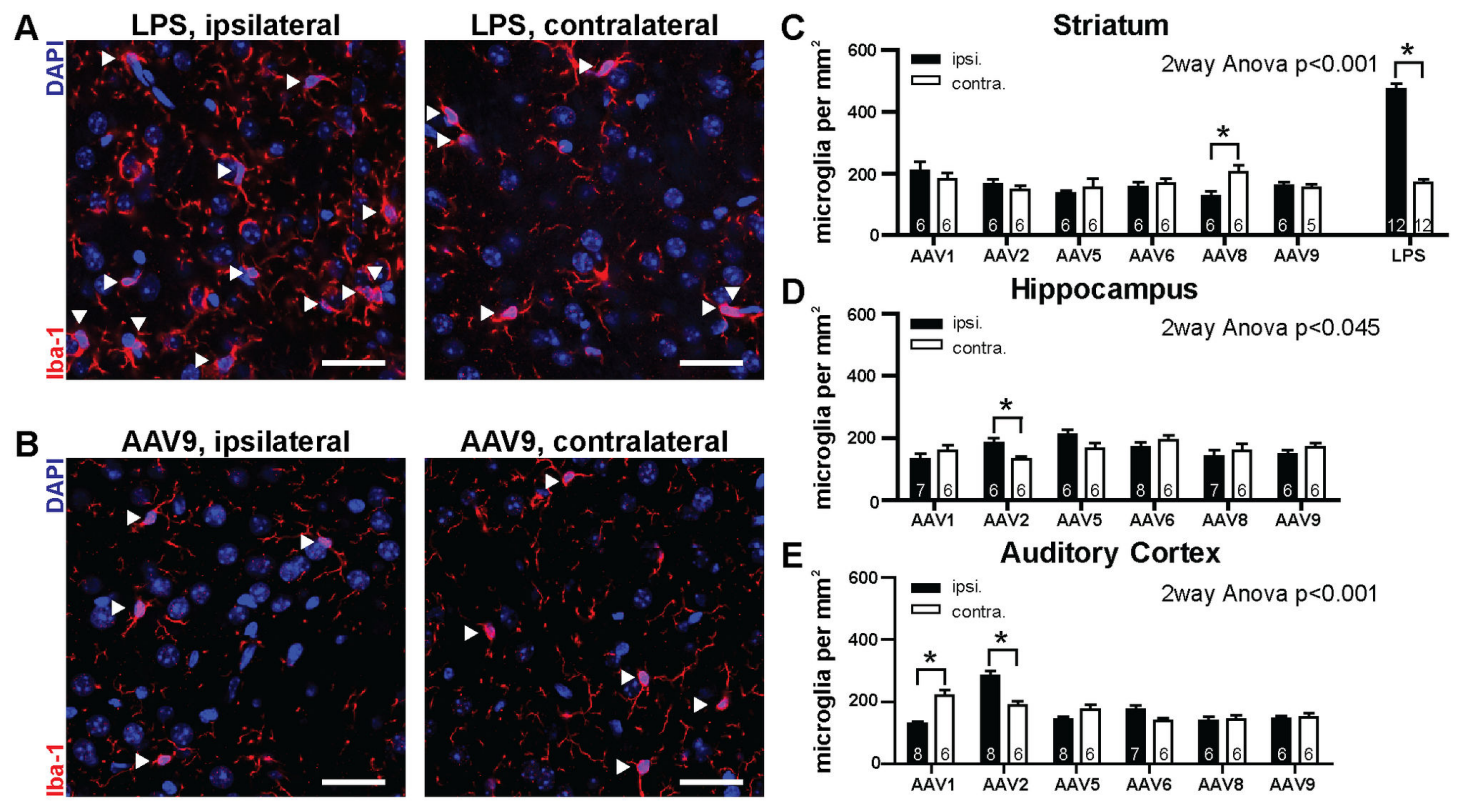
Density of microglia in transduced areas. A) Confocal images taken from the ipsilateral (left) and contralateral (right) striatum of mice that received an injection of LPS. Red channel: immunohistochemical label for Iba1, a marker for microglia. Individual cell bodies of microglia are marked with white arrowheads. Note the high number of microglia in the ipsilateral striatum. B) same as A), however, showing images obtained from mice that received an injection of rAAV9 in the striatum. Note, that microglia counts are low in both hemispheres. Scale bars: 10 µm. C) Quantification of ipsilateral and contralateral microglia densities in the striatum of mice with rAAV and LPS injections. Number of analyzed sections is shown at bottom of bars. D) Ipsilateral and contralateral density of microglia in the hippocampus of mice following rAAV injections. E) Ipsilateral and contralateral density of microglia in the auditory cortex of mice following rAAV injections. All bars represent mean±SEM.

**Figure 5:**
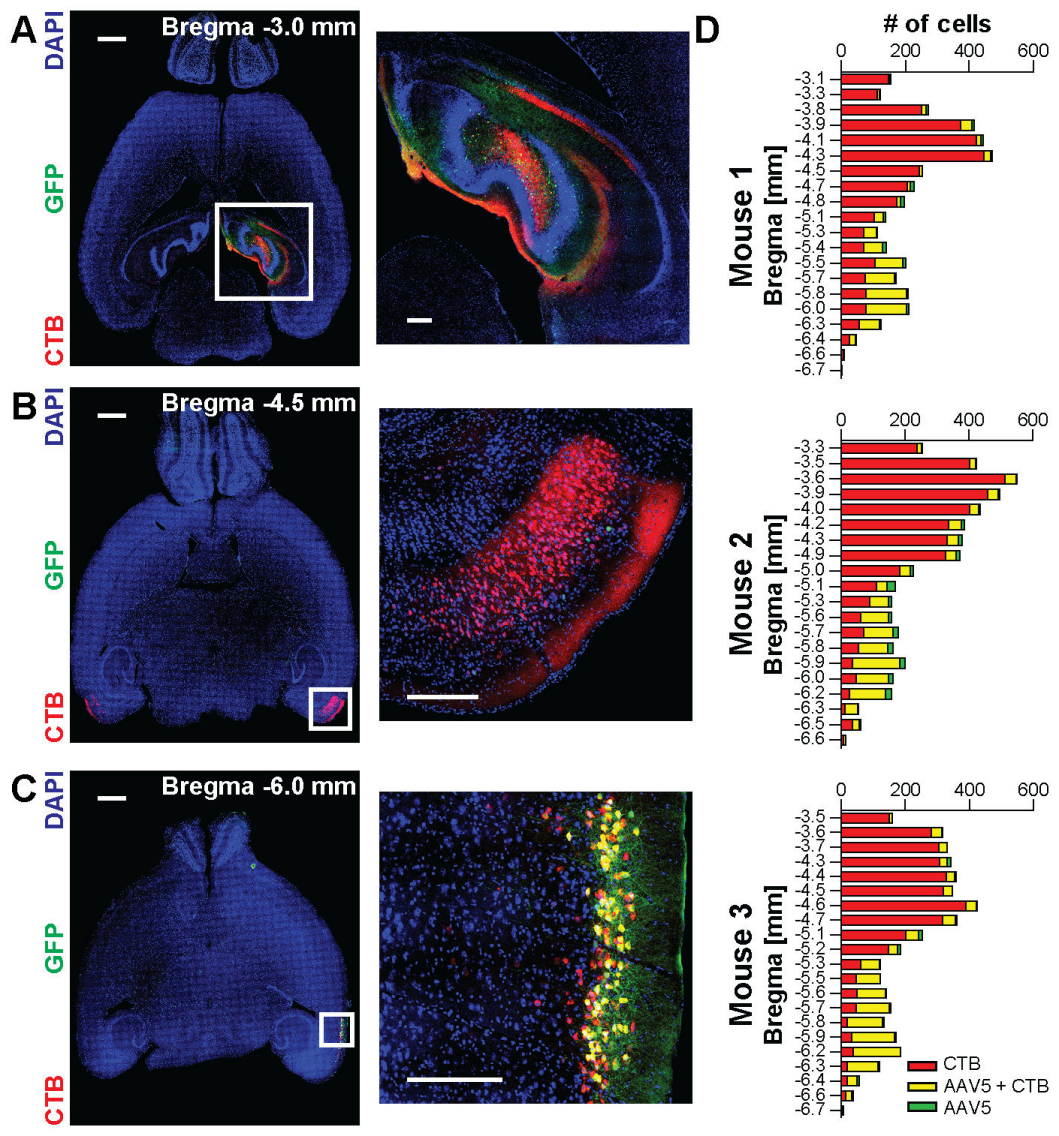
rAAV5 injections in the dentate gyrus lead to retrograde labeling of cells in the ventral entorhinal cortex. A) Confocal images from horizontal brain sections at -3 mm along the dorso-ventral axis in reference to Bregma taken from a mouse that received a co-injection of rAAV5 driving expression of GFP (green) and fluorescently labeled retrograde tracer CTB (red) in the dentate gyrus. B) same as A), for Bregma -4.5 mm. C) same as A), for Bregma -6.0 mm. Double labeled cells in the ventral entorhinal cortex appear yellow. White squares on the left indicate parts of images shown in higher magnification on the right. Scale bars: for whole sections: 1 mm; for insets: 250 µm. D) Cell counts of CTB-only labeled cells (red), GFP-only labeled cells (green) and double positive cells (yellow) in horizontal sections of the ipsilateral entorhinal cortex along the dorso-ventral axis of three individual mice.

**Figure 6:**
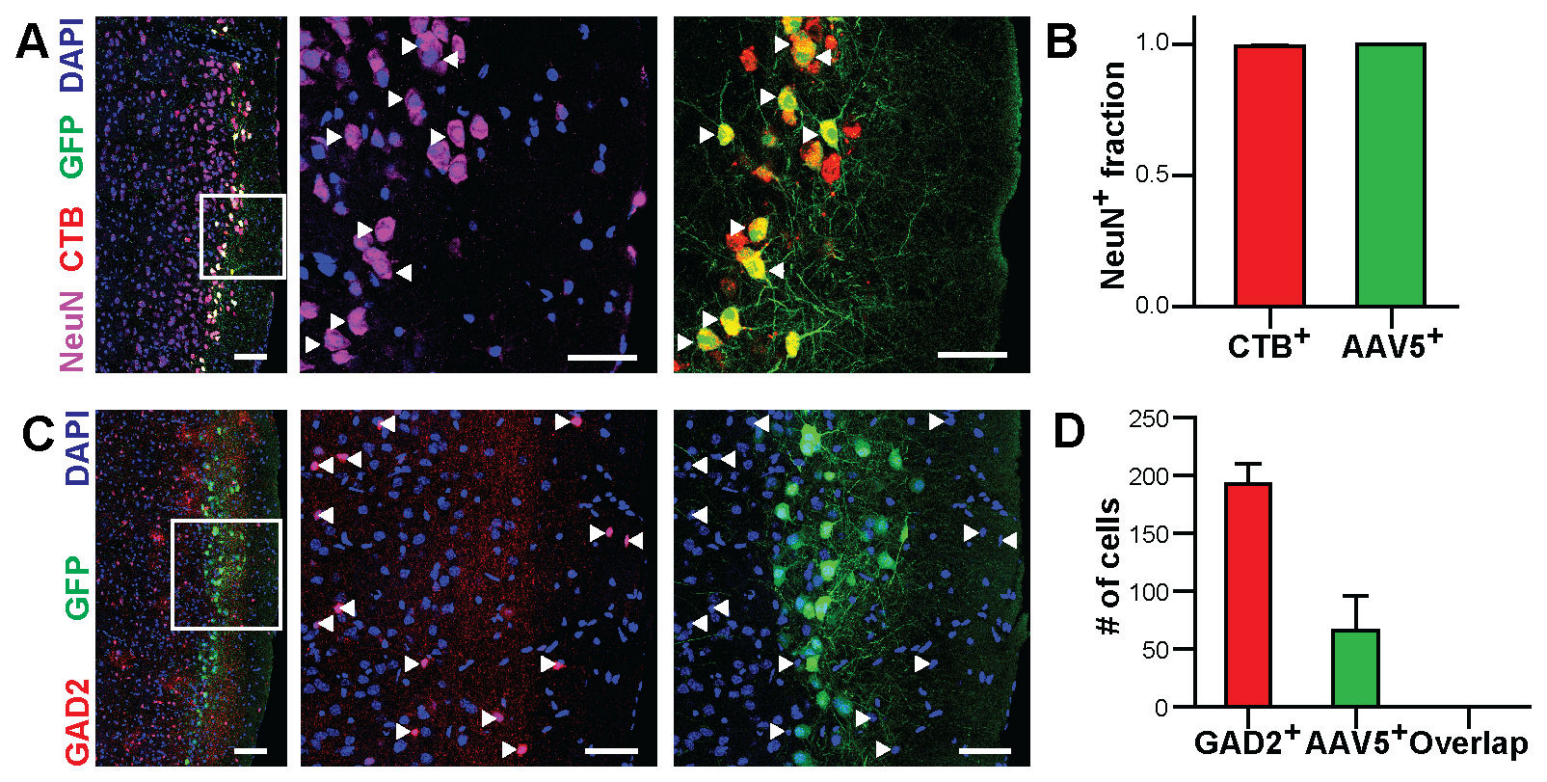
Retrogradely labeled cells in the entorhinal cortex are excitatory neurons. A) Confocal fluorescence images of the same horizontal section of the ventral entorhinal cortex of a mouse that received a co-injection of rAAV5 driving GFP expression (green) and fluorescently labeled CTB (red) that was stained with DAPI (blue) and immunohistochemically labeled against the neuronal marker NeuN (magenta). Examples of individual GFP and CTB-labeled neurons are marked with white arrowheads. The white square denotes the area shown in higher magnification. Scale bars: low magnification: 100 µm, high magnification: 50 µm. B) Quantification of CTB positive cells and GFP positive cells (bars: mean±SEM; CTB: n= 918 cells, 4 sections, one mouse; GFP: n=264 cells, 4 sections, one mouse) with the neuronal marker NeuN showing in both cases almost complete co-labeling. C) Confocal fluorescence images of the same horizontal section of the ventral entorhinal cortex of a transgenic mouse in which GAD2-expressing interneurons are labeled by tdTomato (red) that received an injection of rAAV5 driving GFP expression (green) that was stained with DAPI (blue). Individual interneurons are marked with white arrowheads. The white square denotes the area shown in higher magnification. Scale bars: low magnification: 100 µm and high magnification: 50 µm) D) Quantification of GAD2-expressing interneurons and rAAV5 transduced neurons showing disjunct populations (bars: mean±SEM; n=1033 cells, 4 sections, one mouse).

For a comparison of rAAV vectors derived from different AAV serotypes with respect to transduction efficiency, tropism, microglia induction, and retrograde transport (Figures 1-6) a CMV-EGFP reporter gene cassette was used and inserted into an AAV plasmid containing ITRs derived from AAV2. For this purpose pFBGFPR, a plasmid enabling rAAV production in insect- and mammalian cells [24], was used as an entry vector. Briefly the pCMV/p10-EGFP-SV40pA expression cassette of pFBGFPR was exchanged for a CMV-EGFP expression cassette lacking the p10 promoter sequence by using NotI sites. The resulting plasmid, which was used for production of rAAV1, 2, 5, 6, 8 and 9 virus stocks, was termed pFB-AAV-CMV-EGFP.

**Figure 7:**
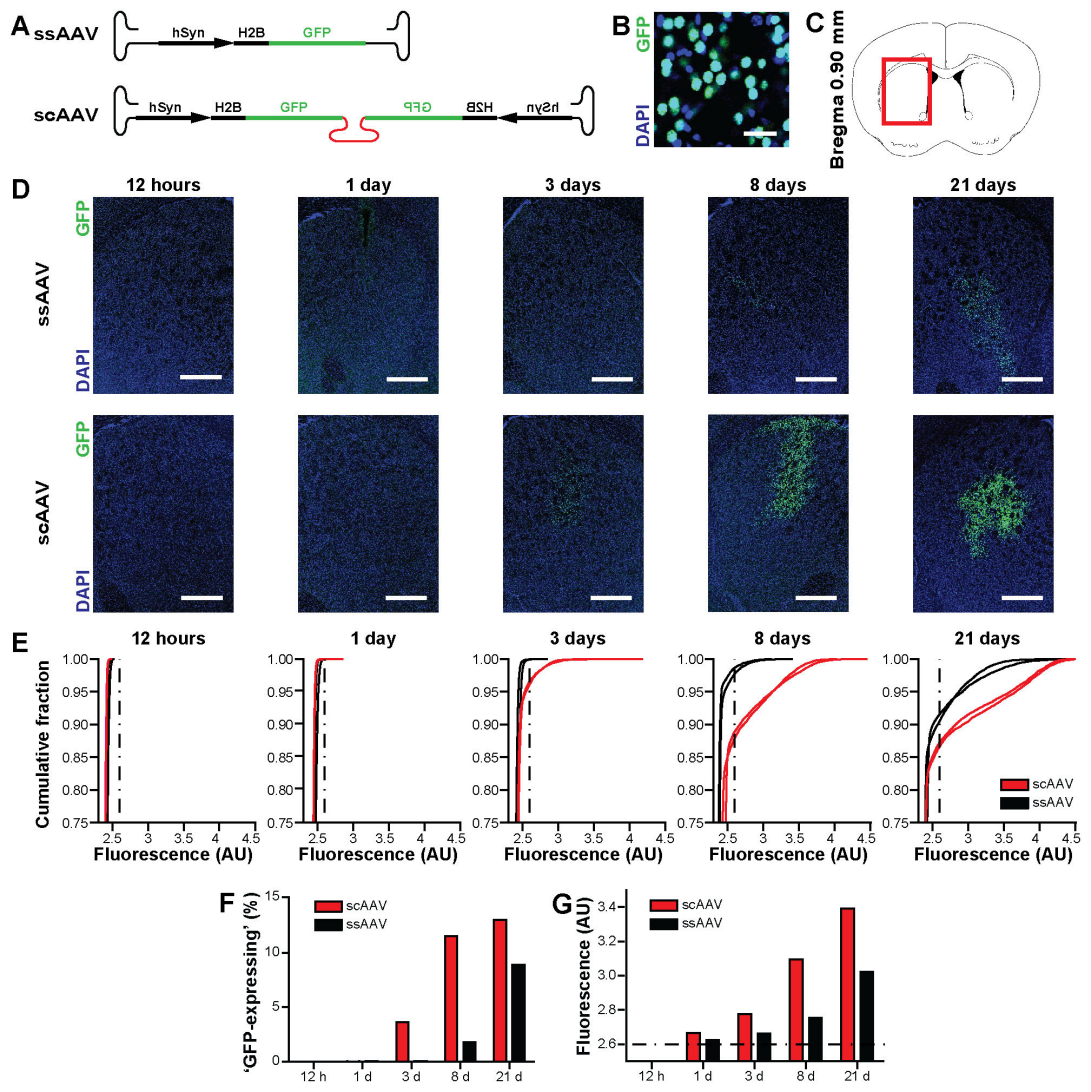
Time course of expression in striatal neurons transduced *in vivo* with ssAAV or scAAV. A) Schematics of the ssAAV and scAAV constructs driving expression of an H2B-GFP fusion protein under the control of the neuronal human Synapsin 1 promoter that have been packaged in AAV8 capsids. B) High-magnification confocal fluorescence image of a coronal striatal section containing scAAV-transduced neurons that was stained with nuclear stain DAPI (blue) showing localization of the H2B-GFP fusion protein (green) in the nucleus. Scale bar: 25 µm. C) Schematic of a coronal brain section in reference to Bregma with a red box indicating the areas shown in D. D) Confocal fluorescence images of coronal striatal sections stained with DAPI (blue) prepared at increasing time points following injection of ssAAV (top row) or scAAV (bottom row). Green GFP expression in neurons becomes visible in ssAAV at eight days, whereas scAAV leads to earlier expression already at three days after injection. Scale bars: 250 µm. E) Cumulative distributions of mean fluorescence in the green channel of individual nuclei identified based on DAPI signal. Each line corresponds to the distribution of a brain section from one injected mouse (region of interest with a fixed size corresponding to approx. 15028±341 (mean±SEM) cells). Fluorescence measurements below 2.6 AU correspond mostly to non-expressing cells (e.g. 12 hour time point), whereas an appreciable fraction of cells in the striatum show intensities well above 2.6 AU as can be seen following longer incubation periods (e.g. 21 day time point). Dashed vertical line corresponds to 2.6 AU threshold. Two sections from two injected mice per virus type were analyzed on each time point. F) Fraction of cells within region of interest that show fluorescence levels higher than 2.6 AU corresponding to classification of ‘GFP-expressing’ (Bars represent mean of two mice). G) Mean fluorescence of all cells with intensities higher than 2.6 AU. Note that not only the number of ‘GFP-expressing’ cells increases with time, but also their intensity levels.

The constructs used to compare the transgene expression kinetics of single strand AAV (ssAAV) and self-complementary AAV (scAAV) (pAAV-phSyn-H2B-EGFP/mCherry; Figure 7 and Figure S4) were cloned the following way: The histone H2B-gene was isolated from mouse cDNA and fused to either EGFP (Addgene plasmid 11153) or mCherry (Addgene plasmid 20938). The fusion protein was cloned into the pAAV-hSyn backbone (Addgene plasmid 26973) using the KpnI and HindIII sites. For the generation of self-complementary AAV plasmids containing phSyn-H2B-EGFP/mCherry expression cassettes pAAV-MCS (Agilent Technologies, catalog no. 240071), a commercially available AAV entry vector, was used and modified as follows: First a β-globin intron sequence was removed by BstBI digestion and subsequent vector religation. In the next step the terminal resolution site of the right ITR was deleted. Therefore the β-globin intron-deleted pAAV-MCS was triple-digested with MscI, EcoRI and PmlI. In two separate ligation steps an MscI/EcoRI fragment (715 bp) was first ligated with an EcoRI/PmlI fragment (567 bp). Afterwards the resulting 1282 bp fragment was inserted into the pAAV-MCS vector backbone derived from a separate MscI digestion, leading to a β-globin intron-deleted self-complementary version of pAAV-MCS (pscAAV-MCS). To finally obtain self-complemetary AAV plasmids containing phSyn-H2B-EGFP/mCherry expression cassettes, phSyn-H2B-EGFP was inserted into pscAAV-MCS via NotI/BbsI. Afterwards the resulting pscAAV-phSyn-H2B-EGFP plasmid was used as a target vector for the insertion of phSyn-H2B-mCherry via ClaI/NsiI. The resulting self-complementary plasmids were analysed for integrity and functionality after packaging into AAV8 by denaturing agarose gel electrophoresis of isolated virus DNA and by comparing transduction kinetics of single stranded and self-complementary rAAV8 virus stocks in Neuro2A cells (Figure S3).

### Packaging/helper plasmids used for rAAV production

For packaging of rAAV constructs containing AAV2-ITRs into capsids derived from AAV1, 2, 5, 6 and 8 commercially available packaging plasmids consisting of AAV2 rep, the respective AAV1, 2, 5, 6 or 8 cap and Ad5 helper functions were used (PlasmidFactory, catalog no. pDPrs1-6 and pDPape8). To enable production of rAAV vectors packaged into AAV9 capsids the cap gene from AAV9 (Accession no. DD233401, pos. 2116-4326) was synthesized (Life Technologies) and subsequently cloned via SwaI/XmaI into pAAV-RC (Agilent Technologies, catalog no. 240071) thereby replacing the AAV2 cap sequence of the original vector.

### Production and purification of rAAV vectors

All rAAV vectors described were produced in HEK 293 cells by using a helper virus free, two-plasmid based production method [25] for rAAV1, 2, 5, 6 and 8 and a three-plasmid based production method for packaging of rAAV9 vectors based on a commercially available AAV helper free system (Agilent Technologies, catalog no. 240071). HEK 293 cells were transfected by using the calcium phosphate method. 72 hours post transfection cells were harvested and collected by centrifugation (2500 x g, 20 min, 4°C). Cell pellets were resuspended in resuspension buffer (50 mM Tris, 2 mM MgCl2, pH 8.5) and lysed by three consecutive freeze/thaw cycles. For removal of genomic DNA cell lysates were incubated with benzonase (50 U/mL) for 1 hour at 37°C. Subsequently rAAV particles were precipitated with CaCl_2_ (25 mM) followed by PEG precipitation (8% PEG-8000, 500 mM NaCl). After resuspension of PEG precipitates in 50 mM HEPES, 150 mM NaCl, 25 mM EDTA, pH 7.4 overnight at 4°C, rAAV particles were further purified by CsCl density gradient centrifugation. Fractions from CsCl density gradients were analyzed by measuring the refractory index. Samples within a refractory index ranging from 1.3774 to 1.3696 were pooled and dialysed against PBS for removal of CsCl by using dialysis cassettes with a molecular weight cutoff of 20 kDa (Thermo Scientific, catalog no. 87738). Finally rAAV preparations were concentrated by using ultrafiltration units with a molecular weight cutoff of 50 kDa (Millipore, catalog no. UFC905024). After addition of glycerol to a final concentration of 10%, rAAV preparations were sterile filtered with Millex-GV filter units (Millipore, catalog no. SLGV013SL), aliquoted, frozen in liquid nitrogen and subsequently stored at -80°C. Genomic titers of purified rAAV stocks were determined by isolation of viral DNA (Viral Xpress DNA/RNA Extraction Reagent, Millipore, catalog no. 3095) and subsequent qPCR analysis using primers specific for CMV- or hSyn-promoter sequences depending on the genomic constructs packaged.

### Animals

Experimental subjects were male CB57BL/6J mice (Charles River – age: 8-12 weeks). In experiments for Figure 6, mice were heterozygous for GAD2-IRES-CRE (Jackson laboratory stock no. 010802 [26]) and the reporter allele ROSA-LSL-tdTOMATO (Allen Institute line Ai9, Jackson laboratory stock no. 007905). All animal experiments were performed in accordance with the Austrian laboratory animal law guidelines for animal research and had been approved by the Viennese Magistratsabteilung 58 (Approval M58/00236/2010/6).

### Stereotaxic injections

For experiments investigating general transduction efficiency three to seven mice were used per serotype and brain region (Figure 1). Animals were deeply anesthetized with a mixture of ketamine and medetomidine (KM; 2.5 mg ketamine-HCl and 0.02 mg medetomidine-HCl/25 g mouse weight) injected intraperitoneally, and positioned in a stereotaxic frame (Kopf Instruments, Tujunga, CA; Stereotaxic System Kopf 1900). A local anaesthetic (lidocaine) was applied subcutaneously before exposure of the skull. Small holes were drilled into the skull and injections were performed unilaterally using a thin glass pipette with 80 nl of virus solution (titer: 9.6 * 10^11^ viral genomes (VG)/ml in PBS) at a flow rate of 20 nl/min (World Precision Instruments, Sarasota, FL; Nanoliter 2000 Injector). Glass pipettes (World Precision Instruments, Sarasota, FL; Glass Capillaries for Nanoliter 2000; Order# 4878) had been pulled with a long taper and the tip was cut to a diameter of 20-40µm. After the injection, the pipette was left in place for 3 minutes, before being slowly withdrawn. Coordinates for injections were (in mm: caudal, lateral, and ventral to bregma): striatum (0.9, 1.5, 3.2), hippocampus (-1.9, 1.6, 1.6), cortex (-2.9, 4.25, 2.5). After surgery, anesthesia was neutralized with 0.02 ml atipamezole. Mice were monitored daily and intraperitoneal injections of carprofen (0.2 ml of 0.5 mg/ml stock) were applied on the first days after surgery.

For injections of LPS (*Escherichia coli 0127:B8*, Sigma-Aldrich, Germany; Figure 4A), mice were anesthetized with 1-2 vol% isoflurane in oxygen and two µl of LPS dissolved in saline (5 µg/µl) were infused at a flow rate of 0.2 µl/min into the striatum (coordinates (in mm) relative to bregma: 0.5, 2.0, -3.5). The cannula was left in place for further 5 minutes before being removed.

In the experiments investigating retrograde transport (Figures 5, 6), three mice were unilaterally injected with 250 nl of a 4:1 mixture of rAAV5 solution (titer s.a.) and cholera toxin subunit B-alexa fluor 555 conjugate (Invitrogen, C-22843; 1 mg/ml in PBS) into the hippocampus (same coordinates as above). Surgery, pharmacology, and injection were carried out as above.

When analyzing the time-course of expression (Figure 7 and Figure S4), mice received 80 nl injections into the striatum (titer: 1.01 * 10^12^ VG/ml; same coordinates as above). One hemisphere was injected with either a (self-complementing) scGFP/scCherry and the other hemisphere was injected with either a (single strand) ssCherry/ssGFP virus solution. Surgery, pharmacology, and injection as above.

### Histological preparation and staining

For the investigation of transduction efficiency (Figure 1), mice were sacrificed 21 days after virus injection, the brains dissected and immersion fixed in 4% PFA overnight. Fixed brains were cut on a vibratome (Leica, VT-1000) to 60 µm thick coronal slices, stained 30 minutes with a 5 mg/l 4', 6-diamidino-2-phenylindole (DAPI) solution and mounted on cover slips. In the experiments analyzing retrograde transport (Figures 5, 6) the same procedure was used, however, brains were cut to 70 µm thick horizontal slices. In the experiments analyzing the cell tropism and microglia induction (Figures 2-4), 21 days (and 48h after LPS injection, respectively) after virus injection mice were anaesthetized with KM (as above) and transcardially perfused with 20 ml PBS containing 10 U/ml Heparin (Sigma, H3393) and 20 ml 4% PFA. Brains were dissected and post-fixed in 4% PFA overnight. Fixed brains were cut on a vibratome to 50 µm coronal slices and incubated for 2h at RT in PBS containing 10% normal goat serum (Jackson Immuno research, 005-000-121) and 1% Triton-X 100 (Sigma-Aldrich, T8787). Subsequently, slices were washed three times for 10 min with PBS and incubated with primary antibody (GABA (Sigma-Aldrich, A2052): 1:1000, NeuN biotin conjugate (Millipore, MAB377B): 1:250, GFAP (Abcam, AB7779): 1:1000, Iba-1 (Wako Chemicals, 019-19741): 1:1000, Olig2 (Chemicon, AB9610): 1:1000)in PBS containing 5% normal goat serum and 0.1% Triton-X 100 at 4 °C overnight. On the next day, the slices were washed three times for 10 min with PBS and incubated with the secondary antibody (Dye-Light 549 goat anti rabbit IgG (Thermo Scientific 35507): 1:1000; streptavidin alexa flour 647 conjugate (Invitrogen, S32357): 1:1000) in PBS containing 5% normal goat serum and 0.1% Triton-X 100 at RT for 2h. Next, slices were washed three times for 10 min with PBS, incubated for 30 min in a 5 mg/l DAPI solution, and mounted on cover slips. Since each section was stained for NeuN, this procedure yields a four color specimen in which nuclei are labeled blue, GFP is visualized in green, neurons labeled in far-red and the respective cell-type marker labeled in red.

For experiments comparing the expression time course of scAAV and ssAAV the same procedure as described above was used, but brains were cut to 70 µm thick coronal slices, and stained 30 minutes with a 1 mg/l DAPI solution.

### Image acquisition and analysis

To analyze general transduction efficacy in various brain regions (Figure 1), for each brain the slice with the largest amount of GFP expression was identified using epifluorescence and a 12 µm thick confocal z-stack was acquired on a LSM510 Axiovert 200M confocal laser scanning microscope (Carl Zeiss) using a 20x/0.6 Plan-Apochromat objective (Carl Zeiss). For image analysis, we used a custom Matlab script in which the single z-plane with the highest amount of GFP fluorescence was identified by quantification of the intensity in the green channel. Next, the background fluorescence of the image was defined as the mean fluorescence in a reference region adjacent to the injection site and subtracted. A fluorescence index was computed for each image by integrating the intensities of all pixels with values larger than five-fold the standard deviation of pixels within the background area. This index is proportional to the amount of GFP molecules in the tissue.

In the experiments analyzing cell-type tropism of various serotypes (Figures 2, 3), confocal images were acquired on a LSM510 microscope using a 40x/1.3 Plan-Neofluar objective (Carl Zeiss). For analysis, a custom script in Metamorph (Molecular Devices, Sunnyvale, CA) was used in which all nuclei of a given cell type in an area of defined size (striatum: 329x329 µm; hippocampus: 307x307 µm; cortex: 307x483 µm) were manually identified using an overlay of the cell-type marker channel with the DAPI channel. For each identified cell a region of interest was defined automatically based on the DAPI signal and the mean intensity in the green channel was quantified, corresponding to the GFP signal in the nucleus. The same procedure was applied in a reference area adjacent to the injection site for GFP-negative nuclei to define the background intensity. For each image the values of nuclei inside the injection site were normalized by division by the mean intensity of negative nuclei.

For the quantification of microglia numbers (Figure 4), confocal images of the ipsilateral and contralateral hemisphere were acquired on a LSM510 microscope using a 40x/1.3 Plan-Neofluar objective (Carl Zeiss). Microglial cells were identified and counted as above.

The analysis of retrograde transport of rAAV5 (Figures 5, 6) was done on 5 µm thick confocal z-stacks that were acquired on a LSM780 microscope (Carl Zeiss) using a 25x/0.8 plan-apochromat and a 40x/1.3 plan-neofluar objective (Carl Zeiss). For quantification, nuclei of GFP-, CTB-, GAD2-, or NeuN-positive and of negative cells were identified and outlined in the maximum intensity projection of the confocal z-stack as described above. A nucleus was considered positive for a given marker if its mean intensity in the given channel was higher than the mean intensity plus five standard deviations of the negative nuclei in the given channel.

To quantify the time course of expression (Figure 7 and Figure S4) the coronal section with the largest amount of GFP or mCherry expression was identified for each brain using epifluorescence. For early time points before the onset of expression, the needle track was used. A confocal image was acquired on a LSM780 microscope (Carl Zeiss) using a 20x/0.8 plan-apochromat objective (Carl Zeiss). For quantification, an area of 5058x3613 µm size and similar location in the striatum was defined and nuclei were identified automatically using the DAPI channel with a custom script in Definiens software suite (Definiens, Carlsbad, CA). The mean intensities in the green and in the red channel for each identified nucleus were computed (15028±341 (mean±SEM) cells per hemisphere).

### Statistical analysis

Results are presented as single observations or as mean±SEM, as indicated in the corresponding figure legends. Statistical analyses were performed using the software GraphPadPrism. To test for differences in transduction efficiency (Figure 1), and in tropism (Figure 3), we used a non-parametric one-way ANOVA with a Kruskal-Wallis test. To compare efficacies across individual serotypes the ANOVA was followed by a Dunn’s multiple comparison post-test. To test for differences in microglial numbers between ipsi- and contralateral hemispheres (Figure 4), we used a two-way ANOVA with a Bonferroni post-test.

## Results

We aimed to quantitatively analyze various AAV serotypes with respect to their characteristics to mediate transgene expression in the mouse brain. Towards this end we packaged the same DNA fragment that was flanked by two ITRs from AAV2 and contained a CMV promoter driving expression of a GFP reporter gene with capsids of serotypes 1, 2, 5, 6, 8 and 9. Packaging was performed in HEK293 cells and virus preparations were purified by PEG precipitation and subsequent CsCl gradient centrifugation (see methods). Titers of genome copies were analyzed using qPCR and were diluted in PBS to 9.6 * 10^11^ viral genomes per ml. We determined the optimal injection volume, ensuring focal expression of the transgene in the targeted brain region, and still providing sufficient dynamic range for quantification of reporter gene expression, by injecting different volumes of rAAV5 virus solution into one of the brain areas to be tested in this study, the striatum. Analysis of reporter gene expression 21 days post-surgery revealed that pressure-injection of 80 nl virus solution through a finely tapered glass capillary leads to focal transduction of solely the targeted brain area without the surrounding tissue within 100-750 µm around the pipette tip while minimizing physical damage potentially caused by the injection (Figure S1). Furthermore, we lower the likelihood of saturation effects that could possibly occlude differences across serotypes. For all experiments deeply anaesthetized mice (8-12 weeks old) were injected. Mice quickly recovered from the injection procedure and we did not observe any obvious changes in their behavior or health status during the time of expression.

### Efficiency of transgene expression in striatum, hippocampus and neocortex mediated by various serotypes

In a first series of experiments we tested the general efficacy of a given serotype to drive expression of a reporter gene in a particular brain area. For each serotype and brain region 3-7 mice were injected and after an incubation period of 21 days their brains were fixated and coronal sections were prepared and stained with 4',6-diamidino-2-phenylindole (DAPI) to reveal the general morphology of the tissue. Confocal images of the DAPI and GFP fluorescence were taken and for each animal the section with the highest level of expression was determined. On that section we calculated the integrated GFP fluorescence of all pixels that were above an intensity threshold (5 standard deviations above background fluorescence) which resulted in a metric that is proportional to the amount of GFP molecules that have been produced from the viral vectors. We observed detectable GFP expression for all serotypes in all brain regions and that injections of the same serotype and brain region led to reproducible fluorescence levels (average CV across serotypes and brain regions: 0.53; Figure 1). The levels of fluorescence varied across the different brain regions, likely due to differences in the cell density and cell types. Also differences between serotypes within the same brain region could be observed (striatum: one-way ANOVA p < 0.008; hippocampus: one-way ANOVA p < 0.001; auditory cortex: one-way ANOVA p < 0.02). The ranking of serotypes with respect to their ability to drive transgene expression was largely similar across analyzed brain regions with rAAV5, 8 and 9 displaying good expression levels and rAAV2 in all regions with the lowest level of expression. Striatum: AAV5>AAV9>AAV8>AAV1>AAV6>AAV2; hippocampus: AAV9>AAV5>AAV8>AAV1>AAV6>AAV2; auditory cortex: AAV9>AAV8>AAV6>AAV5>AAV1>AAV2.

### Analysis of cell-type tropism for various serotypes

Tissue of the central nervous system is highly heterogeneous and consists of many different cell types including excitatory and inhibitory neurons as the principal neuronal cell classes. In addition, various types of glia cells modulate neuronal function, secure energy metabolism and can mediate immune responses among other functions. To analyze the transduction efficacy for five major cell-types we combined the injection of the various serotypes with subsequent immunohistochemical detection of specific marker proteins. We chose gamma-aminobutyric acid (GABA) as a marker for the major population of inhibitory neurons in the central brain [27], oligodendrocyte transcription factor 2 (Olig2) as a marker for oligodendrocytes [28], ionized calcium-binding adapter molecule 1 (Iba1) as a marker for microglia [29] and glial fibrillary acidic protein (GFAP) for astroglia [30]. In addition, all sections were stained with the pan-neuronal marker neuronal nuclei (NeuN) [31] and the DNA stain DAPI that highlights nuclei. High-resolution confocal images were taken in four fluorescent channels of the DAPI signal, the NeuN signal, the GFP signal and the signal corresponding to the particular cell type marker (Figure 2A, 2C and Figure S2). 4-22 images from one mouse per serotype and brain region were taken from the focus of transduction, i.e. from a field of view close to the injection site that showed an even distribution of transduced cells. Qualitatively, we observed that it was possible to drive GFP expression in all cell types analyzed with each of the injected serotypes. This is consistent with the observation that AAV has a generally broad tropism for many different cell types [32,33].

To quantify GFP expression levels in a particular cell type across serotypes we measured the GFP signal in the nuclei of individual cells identified based on the specific marker and the DAPI signal inside the nucleus. The individual GFP measurements in transduced cells were normalized to the average intensity level of non-transduced cells of the same cell type. Here, we observed that the expression levels of individual cells varied considerably, as can be seen by the spread of the cumulative distributions of the individual cell measurements obtained from a given image (Figure 2B, 2D). Furthermore, we observed systematic differences between serotypes. This is reflected by consistent shifts in the median fluorescence values for various serotypes (intersection with the 0.5 line in the cumulative distributions). For example, we found that astrocytes in the hippocampus showed much higher expression following transduction with rAAV8 as compared to all other serotypes including rAAV5 (Figure 2B). Also, neurons in the cortex displayed higher GFP levels after transduction with rAAV9 as compared to rAAV1 (Figure 2D). In Figure 3 the full set of the average median fluorescence values for all combinations of serotypes, cell types and brain regions is shown.

### Analysis of microglia in transduced tissue

The usefulness of viruses as a gene delivery tool can be strongly limited when the viral infection per se leads to a significant alteration of the normal function of the tissue under observation. Such effects can include necrosis and the induction of immune responses. AAV is generally known for low levels of immunogenicity [8,9], however, a systematic comparison across serotypes in the brain is lacking. We therefore sought to characterize the injection sites of various serotypes with respect to markers of immune responses. One of the analyzed cell types above is microglia, which we detected based on the label of the macrophage/microglia specific EF hand protein Iba1. It is known that the number of microglia cells is dramatically increased in inflamed and necrotic tissues [34,35]. Consistent with these previous findings, we observed a strong, ipsilateral increase of Iba1-positive cells in brain slices from mice that had received an injection of lipopolysaccharide (LPS) in the striatum 48h earlier. This stimulus is known to induce an acute inflammatory response [36] and shows that our protocol to detect microglia is sensitive to tissue undergoing an immune reaction and that this reaction is limited locally to one hemisphere (Figure 4). Based on this conclusion we went on and counted Iba1-positive cells of mice injected with various rAAV serotypes in an ipsilateral region of interest containing the injection site and a corresponding contralateral region of interest of the same size (Figure 4A). When considering numbers of Iba1-positive cells in mice that had received injections of rAAV at the time point of expression analysis (three weeks after injection), we generally observed microglia counts about three-fold lower as compared to the acute LPS response. The statistical analysis revealed significant differences in expression levels in all three brain areas (Striatum: two-way ANOVA p<0.001; Hippocampus: two-way ANOVA p<0.045; Auditory Cortex: two-way ANOVA p<0.001). However, these differences were rather small and did not reflect systematic increases in microglia number in the injected hemisphere. They are possibly due to sources of variability other than the injected viruses. In summary, these results indicate that transduction of brain tissue with the analyzed rAAV serotypes does not lead to a chronic induction of an immune response at levels comparable to what can be observed following LPS injections, which is consistent with previous reports of low cytotoxicity *in vitro* [37]. In this study we analyzed microglial numbers at one time point, 21 days after application, and therefore possible short- or long-term effects cannot be excluded. A more detailed analysis of potential immunogenicity could be complemented by measurements of pro-inflammatory factors (e.g. TNFα, MCP-1, IL-1β, or NF-κB p65).

### Retrograde axonal transport of rAAV5 in excitatory neurons projecting from the entorhinal cortex to the dentate gyrus

Viruses enter their host cells at specific sites at the cell surface and need to be transported to the nucleus prior to replication. In the case of neurons featuring widespread arborizations specific entry and intracellular transport can be used for tracing of neuronal connections within brain circuits. Some viruses, including certain herpes virus strains, allow retrograde tracing of neuronal projections due to axonal entry and retrograde transport along the axon [38,39]. Others, including rabies virus, allow even transport across synapses, and thus the ability to trace synaptically connected neurons [40-43]. In the case of rAAV most serotypes are reported to transduce primarily cells close to the injection site, which does not indicate specific uptake and transport mechanisms involved in the rAAV-mediated transduction. However, recent publications indicate a potential for transport in the nervous system for specific serotypes [44-46]. To address the point of transport of rAAV serotypes in the central nervous system in our study, we investigated coronal brain sections up to 1 mm distant along the anterior-posterior axis of the primary transduction focus. In particular, we searched for the presence of GFP-labeled cell bodies that are clustered at distances more than 500 µm from the injection site that could not be explained by simple diffusion of the virus solution. We could not detect signatures for transport in the nervous system in our samples except for one experimental condition: injection of rAAV5 in the dentate gyrus led to a clear and reproducible labeling of cell bodies in the ipsilateral lateral entorhinal cortex and to lesser extent also in the contralateral lateral entorhinal cortex in all injected mice (Figure 5; n=9). The entorhinal cortex is known to project extensively to the dentate gyrus [47]. To test if GFP labeling is indeed due to specific retrograde axonal transport of AAV5, we co-injected rAAV5 driving GFP expression with the established retrograde tracer Cholera Toxin B (CTB; [48]), which was conjugated to a fluorescent dye (alexa-fluor 555). In all three injected mice GFP expression was almost exclusively observed in cells that were also labeled by CTB. Intriguingly, the rAAV5 labeled cells were most frequent in layer 2 of the ventral part of the lateral entorhinal cortex potentially labeling a distinct sub-population of projection neurons (Figure 5D). In addition, we performed immunohistochemical detection of the neuronal marker protein NeuN in brain slices prepared from CTB and rAAV5 co-injected mice and found that nearly all CTB positive cells and nearly all rAAV5 positive cells were also stained positive for NeuN (n=1182 cells from 4 slices from one mouse; Figure 6A, 6B). This result further corroborates the case of a specific transport of rAAV5 in neurons, since non-specific spread of the virus solution along myelin bundles would have likely led to labeling of also other cell types in the entorhinal cortex, similar as we have observed in proximity to the injection site. Long-range projections between parts of the brain are primarily mediated by excitatory neurons. However, a specific population of GABA-ergic neurons was recently described in the entorhinal cortex and the dentate gyrus that features reciprocal connections [49]. To determine what population of long-range projecting neurons is selectively labeled by AAV5 we performed injections of rAAV5 in a transgenic mouse strain in which GABA-ergic interneurons are labeled by the red fluorescent reporter protein tdTomato [26,50]. Here, we again quantified the overlap of GFP-expressing and tdTomato expressing neurons in the entorhinal cortex, but could not find double-labeled cells (n = 1033 cells from 4 slices from one mouse; Figure 6C, 6D). This indicates that rAAV5-mediated retrograde labeling occurs selectively at excitatory connections from the lateral entorhinal cortex to the dentate gyrus. The transport appears highly selective since we did not observe distally labeled cell bodies in other parts of the brain of rAAV5 transduced mice where dense projections exist, e.g. reciprocal callosal projections between auditory cortices. The transport appeared even specific within the broader population of retrogradely labeled neurons in the entorhinal cortex, where essentially only the ventral proportion of CTB-labeled neurons showed AAV5-mediated GFP fluorescence.

### Time-course of transgene expression mediated by single-stranded and self-complementing rAAV

rAAV-mediated gene delivery offers many advantages, however, in experimental conditions where a quick onset of expression is crucial, other virus systems like Herpes amplicons, Sindbis or viruses that allow expression within hours to few days may be the preferred choice [51-54]. It is believed that a rate-limiting step for the AAV-mediated expression of transgenes is the formation of double-stranded DNA [55]. Recent reports demonstrated the usage of rAAV constructs with a self-complementing structure (scAAV) in which the two halves of the single-stranded AAV genome can form an intra-molecular double-strand. This approach reduces the effective genome size usable for gene delivery to about 2.3kB, but leads to significantly shortened onsets of expression in comparison with conventional single-stranded AAV expression constructs (ssAAV) [56]. Self-complementing AAVs have been utilized to transduce cultures from primary cells and multiple tissues after systemic injection, including the brain [57-59]. It remains unknown within what timescale transgene expression can be driven using scAAV in the central nervous system *in vivo*.

To characterize the time-course of rAAV-mediated expression in the mouse brain following scAAV and ssAAV transduction, we cloned constructs containing AAV2 ITRs in which expression of an H2B-GFP fusion protein is driven from the neuron-specific promoter of the human synapsin 1 gene leading to fluorescent labeling of the nuclei of neurons (Figure 7A, 7B). One construct was designed in the conventional way. The self-complementing construct was designed by deleting the terminal resolution site (trs) within the right ITR as described in the methods section. We packaged the two constructs in AAV8 capsids and injected 80 nl containing 8.1 * 10^7^ genome copies in the striata of mice (Figure 7C). Mice were sacrificed at 0.5, 1, 3, 8 and 21 days following injection and brain slices containing the striatum were counter-stained with DAPI. We acquired confocal images in the green and blue fluorescence channel with identical settings (Figure 7D). We analyzed two injections per virus construct and time point corresponding to 15028±341 (mean±SEM) cells each, in a region of interest containing the transduction focus itself and a significant proportion of surrounding non-transduced tissue. The pipette track was used as an indicator of the injection site for early time points when fluorescence was not yet detectable. We quantified the fluorescence in the green channel of nuclei that had been detected based on the DAPI stain. The cumulative distributions of fluorescence measurements of individual cells are tight and show low intensity values for most cells around 2.4 AU. In the case of ssAAV only after 8-21 days following injection a fraction of 2-9% of cells show fluorescence levels higher than 2.6 AU, which we defined as ‘GFP-expressing’ (Figure 7F). However, for mice injected with scAAV an appreciable number of cells showing GFP expression could be detected already after 3 days, with a further increase in cell number after 8 and 21 days (Figure 7F). We furthermore quantified the mean fluorescence of all cells with fluorescence levels higher than 2.6 AU (Figure 7G). We found that with scAAV the number of cells only slightly increased from 8 to 21 days, but the levels of GFP still further increased. At all time points the median fluorescence of transduced cells was higher in case of scAAV as compared to ssAAV. We observed similar results in a complementing set of experiments in which similar scAAV and ssAAV constructs have been used in which the fluorescent protein GFP was replaced by mCherry (Figure S4).

## Discussion

In this study we comprehensively analyzed the transduction properties of rAAV vectors based on different AAV serotypes in the mouse brain to provide a detailed dataset that allows direct cross-comparisons and thus can be used to choose an optimal vector for gene delivery in the mouse central nervous system. For the first time, we included quantitative measures of transduction efficacies and an in-depth analysis of cell-type tropisms using a combination of immunohistochemical labeling of cell-types and virus injections in histological samples. Consistent with previous studies, we observed that individual serotypes differ strongly in their effectiveness to drive gene expression in the mouse brain [12,16,17]. The general profile in efficiency across serotypes is largely similar in the investigated brain areas, whereas rAAV5 was most effective in the striatum, but less so in the cortex, where rAAV9 was most effective. It turns out that AAV2, one of the earliest used serotypes for gene delivery in the brain was least effective.

Co-labeling experiments for cell-type specific markers revealed GFP expressing cells of all cell-types following transduction with any of the analyzed serotypes. This indicates that rAAV vectors have a generally broad potential to transduce cells within the brain. In our study, we focused on a quantitative side-by-side analysis of different serotypes to transduce a given cell type, as the readout of the reporter could be affected by other factors than the serotype when comparing across cell types. GFP levels are determined by the efficacy of the CMV promoter in a given cellular context, the morphology of the cell and other factors [60]. If particular cell-types need to be targeted, specific promoters can be used to restrict expression of the transgene [61]. Alternatively, Cre-recombinase dependent constructs could be used in the background of Cre-expressing driver mouse lines [62]. Despite the broad transduction potential, we observed marked differences in the expression levels that can be achieved with specific serotypes in selected cell types. For example, rAAV8 was highly efficient in driving GFP expression in astrocytes or rAAV9 in cortical neurons. This could be due to higher concentrations of serotype-specific cellular receptors on certain cell-types [63-65]. Thus, if experimental requirements demand particularly high expression levels in a chosen cell type or maximal coverage within a region, it is advisable to choose a particularly effective serotype.

Transport of viruses within the nervous system can be of interest for tracing studies and projection-specific genetic targeting of neurons [2]. Few studies report indication for axonal or trans-synaptic transport for AAV [44-46]. In our study we primarily observed transduced cell bodies only in the immediate vicinity of the injection site independent which serotype was injected in any of the three analyzed brain areas. However, we observed one exemption: injection of rAAV5 in the dentate gyrus led to transduction of excitatory neurons in the lateral entorhinal cortex via retrograde axonal transport, as the co-labeling with CTB indicated. This observation indicates that this mode of transport appears to be highly selective: First, we did not observe axonal transport with other serotypes than rAAV5. Second, we did not observe retrograde axonal transport from the dentate gyrus to other brain areas with known anatomical projections. Third, retrograde transport of rAAV5 was selective for a subpopulation of CTB labeled neurons in the lateral entorhinal cortex. The platelet-derived growth factor receptor (PDGFR) was identified as a receptor participating in AAV5-mediated transduction [66]. However, in situ expression data does not indicate selective expression of PDGFR alpha or beta in the entorhinal cortex that could explain specific retrograde labeling of entorhinal neurons in a simple manner [67]. Therefore further work is required to identify the molecular mechanisms mediating this specificity. For example, trafficking of PDGFR to the axon terminals or factors controlling the transport within the cell along the axon could be regulated in a cell-type specific manner. Identification of these molecular determinants holds the promise to develop approaches that would allow specific targeting of projections other than the one identified in this study or the development of generally retrogradely transported AAV variants for the central nervous system. However, in case a locally restricted targeting is desired, it is advisable to perform a careful histological analysis to exclude possible retrograde transport to other brain regions when rAAV5 is considered for optogenetic or pharmacogenetic manipulations. Recently, retrograde labeling of entorhinal neurons has been also reported following hippocampal injections of rAAV1 [68]. In this study multiple injections with significantly larger amounts of viruses have been used to achieve retrograde labeling. This could indicate that, similar to the ability to transduce a given cell type, different serotypes may have different efficacies in axonal transport and that this could be partially compensated by the viral dosage. With the relatively low viral amounts used in our study, we likely were sensitive to detect only axonal transport that was particularly efficient.

AAV-mediated gene transfer is characterized by a relatively slow onset of expression compared to other viruses like Herpes or Sindbis [51-54]. Our analysis demonstrates that the usage of scAAV is an effective strategy to shorten the time required for expression to few days rather than weeks and to enhance effective expression levels *in vivo*. Although we demonstrated the effects of self-complementation on the expression time in rAAV8, similar observations are expected independent of the capsid used. A general aspect of rAAV mediated gene transfer is that expression levels of the introduced transgene can be highly variable in individual cells of the transduced population (Figure 7E and Figure S4E).

In summary, we here provide a quantitative dataset to choose a suitable AAV serotype for various applications to target specific regions and cell types in the mouse brain.

## Supporting Information

Figure S1 **Expression of GFP following striatal injections of various amounts of rAAV5.** We injected various volumes of rAAV5 (9.6*10^11^ VG/ml) driving expression of GFP from a CMV promoter into the striatum and analyzed transduction efficacy after 21 days of incubation. A) Schematic of a coronal brain section in reference to Bregma with a red box indicating the areas shown in B. B) Confocal images of brain sections of the left hemisphere stained with DAPI (blue) showing expression of the reporter GFP (green). Injection of larger volumes leads to increased GFP expression. An injection volume of 80 nl does not lead to saturation of expression. Note, injections of large volumes cause massive transduction also outside of the striatum. Scale bars: 1 mm. C) Mean fluorescence index (see methods) calculated for a region of interest encompassing the striatum following transduction with different volumes. The images for this series of experiments were acquired with different settings as those shown in Figure 1A to avoid saturation of the GFP signal in mice injected with large volumes, thus the fluorescence index is not directly comparable to Figure 1A. Four hemispheres were injected and analyzed per volume. All bars represent mean±SEM. Asterisk indicates significant differences at the p<0.05 level.
(TIF)

Figure S2 **Immunohistochemichal identification of five different cell types.** A) Confocal images of brain sections of the auditory cortex prepared from mice that received injections of AAV driving GFP expression under the control of the CMV promoter. Red channel: immunohistochemical label for Olig2, an oligodendrocyte specific transcription factor; blue channel: DAPI, labeling nuclei; green channel: GFP expression driven by viral vector. Examples of individual immunohistochemically identified cell bodies are marked with white arrowheads. B) Same as in A) with the immunohistochemical label for Iba1, a microglia specific cytosolic marker. C) Same as in A) with the immunohistochemical label for GFAP, an astrocyte specific intermediate filament. D) Same as in A) with the immunohistochemical label for NeuN, a neuron specific splicing factor. E) Same as in A) with the immunohistochemical label for GABA, an inhibitory neuron specific neurotransmitter. Scale bars: 25 µm.
(TIF)

Figure S3 ***In vitro* characterization of single stranded and self-complementary AAV8-hSyn-mCherry/GFP virus stocks.** A) Viral vector DNA was isolated from purified AAV8 virus stocks and subsequently loaded on a denaturing alkaline agarose gel for size analysis. For single stranded vector genomes (ssAAV8) expected band sizes of 2422 bp (mCherry) and 2431 bp (GFP) were confirmed, whereas a doubling in band size to approximately 4.8 kb was observed for the respective self-complementary vector genomes (scAAV8) as expected. In samples from ssAAV8 virus stocks additional bands were detected at 4.8 kb as indicated by asterisks (*). These bands most likely originate from a subfraction of ssAAV8 particles containing dimeric genomes due to imperfect terminal resolution at the wtITR, a phenomenon frequently observed for packaging of small vector genomes with sizes below 2.5 kb. B) Neuro2A cells were transduced with single stranded and self-complementary AAV8-hSyn-mCherry/GFP virus stocks at an MOI of 2,5x10^5^ vector genomes per cell. 72 hours post transduction expression of fluorescent reporter genes was assessed by fluorescence microscopy, demonstrating superiority of scAAV8 virus stocks compared to ssAAV8 virus stocks as indicated by increasing numbers in GFP/mCherry-positive cells and enhanced transgene expression levels. Scale bars: 50 µm.
(TIF)

Figure S4 **Time course of expression in striatal neurons transduced *in vivo* with ssAAV or scAAV coding for a red fluorescent protein.** The experiment and analysis is analogous to the one shown in Figure 7, the only difference is that H2B was fused to mCherry instead of GFP. A) Schematics of the ssAAV and scAAV constructs driving expression of an H2B-mCherry fusion protein under the control of the neuronal human Synapsin 1 promoter that have been packaged in AAV8 capsids. B) High-magnification confocal fluorescence image of a coronal striatal section containing scAAV-transduced neurons that was stained with nuclear stain DAPI (blue) showing localization of the H2B-mCherry fusion protein (red) in the nucleus. Scale bar: 25 µm. C) Schematic of a coronal brain section in reference to Bregma with a red box indicating the areas shown in D. D) Confocal fluorescence images of coronal striatal sections stained with DAPI (blue) prepared at increasing time points following injection of ssAAV (top row) or scAAV (bottom row). Red mCherry expression in neurons becomes visible in ssAAV at eight days, whereas scAAV leads to earlier expression already at three days after injection. Scale bars: 250 µm. E) Cumulative distributions of mean fluorescence in the red channel of individual nuclei identified based on DAPI signal. Each line corresponds to the distribution of a brain section from one injected mouse (region of interest with a fixed size corresponding to approx. 15028±341 (mean±SEM) cells). Fluorescence measurements below 2.6 AU correspond mostly to non-expressing cells (e.g. 12 hour time point), whereas an appreciable fraction of cells in the striatum show intensities well above 2.6 AU as can be seen following longer incubation periods (e.g. 21 day time point). Dashed vertical line corresponds to 2.6 AU threshold. Two sections from two injected mice per virus type were analyzed on each time point. F) Fraction of cells within region of interest that show fluorescence levels higher than 2.6 AU corresponding to classification of ‘mCherry-expressing’ (Bars represent mean of two mice). G) Mean fluorescence of all cells with intensities higher than 2.6 AU. Note that not only the number of ‘mCherry-expressing’ cells increases with time, but also their intensity levels.
(TIF)
