# Supplementary figures and images for: Analysis of Transduction Efficiency, Tropism and Axonal Transport of AAV Serotypes 1, 2, 5, 6, 8 and 9 in the Mouse Brain
Source: PLoS One. 2013 Sep 27;8(9):e76310. doi: 10.1371/journal.pone.0076310 (PMC3785459; doi:10.1371/journal.pone.0076310)

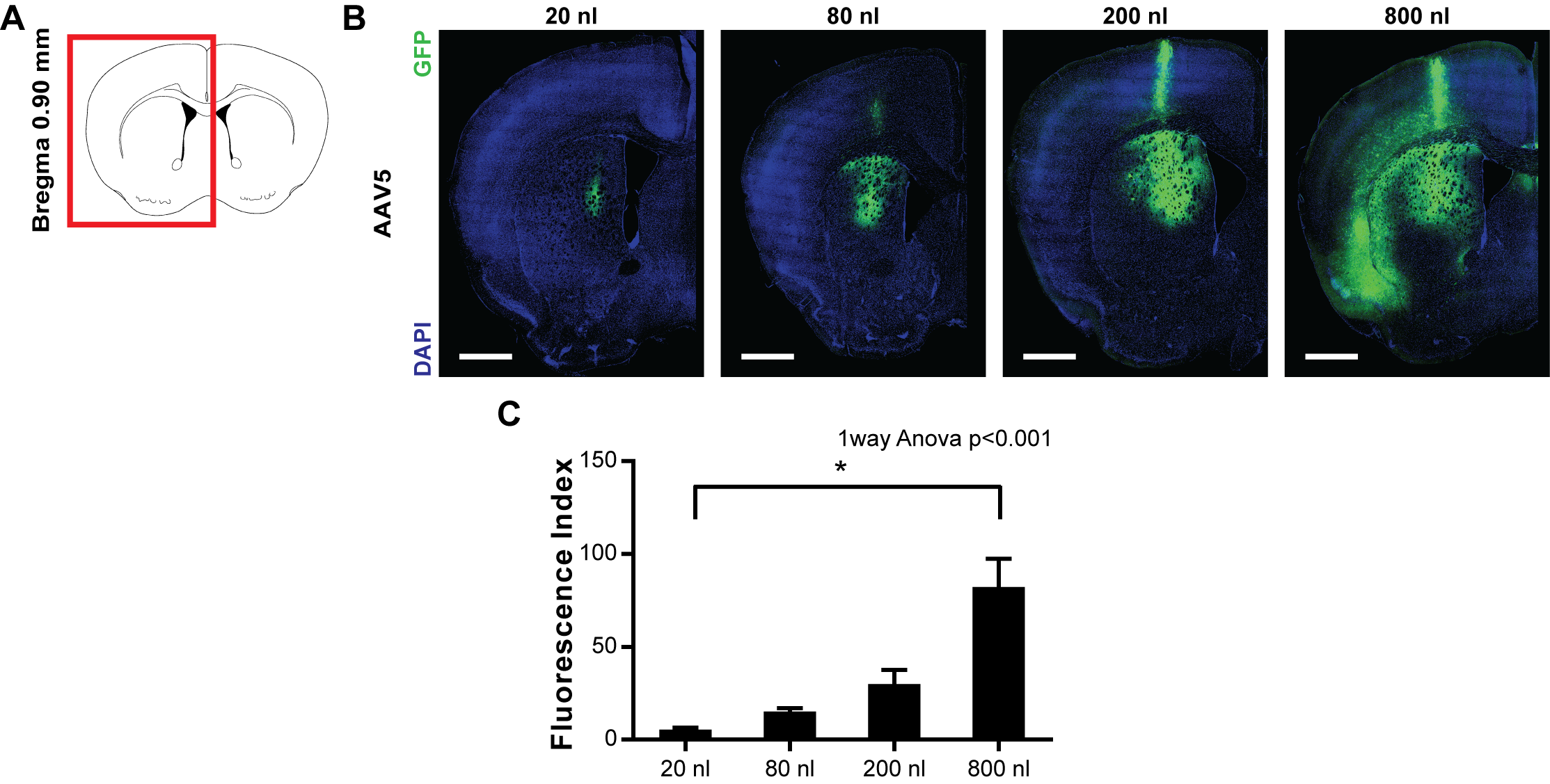

Supplement: Figure S1 — Expression of GFP following striatal injections of various amounts of rAAV5. We injected various volumes of rAAV5 (9.6*1011 VG/ml) driving expression of GFP from a CMV promoter into the striatum and analyzed transduction efficacy after 21 days of incubation. A) Schematic of a coronal brain section in reference to Bregma with a red box indicating the areas shown in B. B) Confocal images of brain sections of the left hemisphere stained with DAPI (blue) showing expression of the reporter GFP (green). Injection of larger volumes leads to increased GFP expression. An injection volume of 80 nl does not lead to saturation of expression. Note, injections of large volumes cause massive transduction also outside of the striatum. Scale bars: 1 mm. C) Mean fluorescence index (see methods) calculated for a region of interest encompassing the striatum following transduction with different volumes. The images for this series of experiments were acquired with different settings as those shown in Figure 1A to avoid saturation of the GFP signal in mice injected with large volumes, thus the fluorescence index is not directly comparable to Figure 1A. Four hemispheres were injected and analyzed per volume. All bars represent mean±SEM. Asterisk indicates significant differences at the p<0.05 level. (TIF) [file pone.0076310.s001.tif]

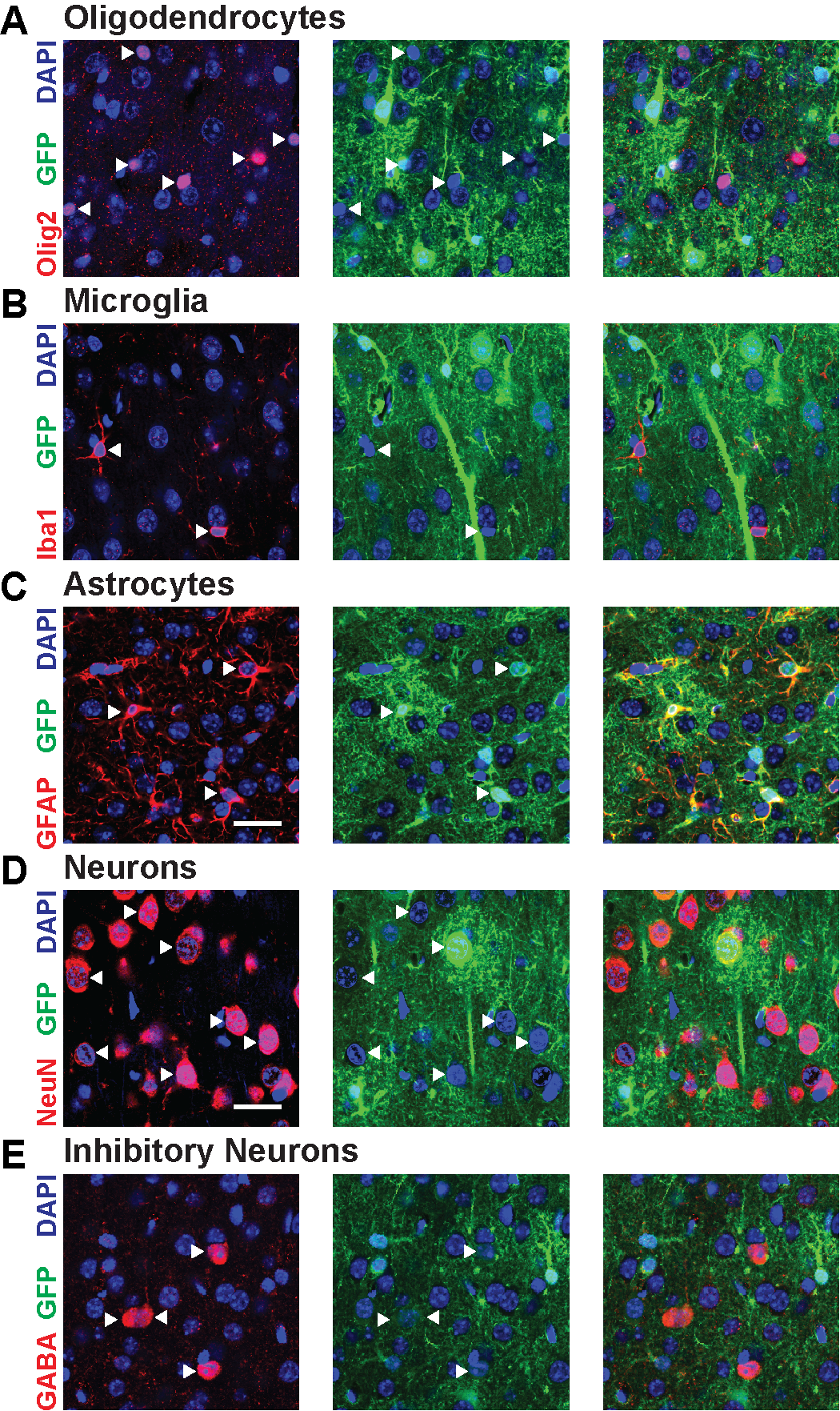

Supplement: Figure S2 — Immunohistochemichal identification of five different cell types. A) Confocal images of brain sections of the auditory cortex prepared from mice that received injections of AAV driving GFP expression under the control of the CMV promoter. Red channel: immunohistochemical label for Olig2, an oligodendrocyte specific transcription factor; blue channel: DAPI, labeling nuclei; green channel: GFP expression driven by viral vector. Examples of individual immunohistochemically identified cell bodies are marked with white arrowheads. B) Same as in A) with the immunohistochemical label for Iba1, a microglia specific cytosolic marker. C) Same as in A) with the immunohistochemical label for GFAP, an astrocyte specific intermediate filament. D) Same as in A) with the immunohistochemical label for NeuN, a neuron specific splicing factor. E) Same as in A) with the immunohistochemical label for GABA, an inhibitory neuron specific neurotransmitter. Scale bars: 25 µm. (TIF) [file pone.0076310.s002.tif]

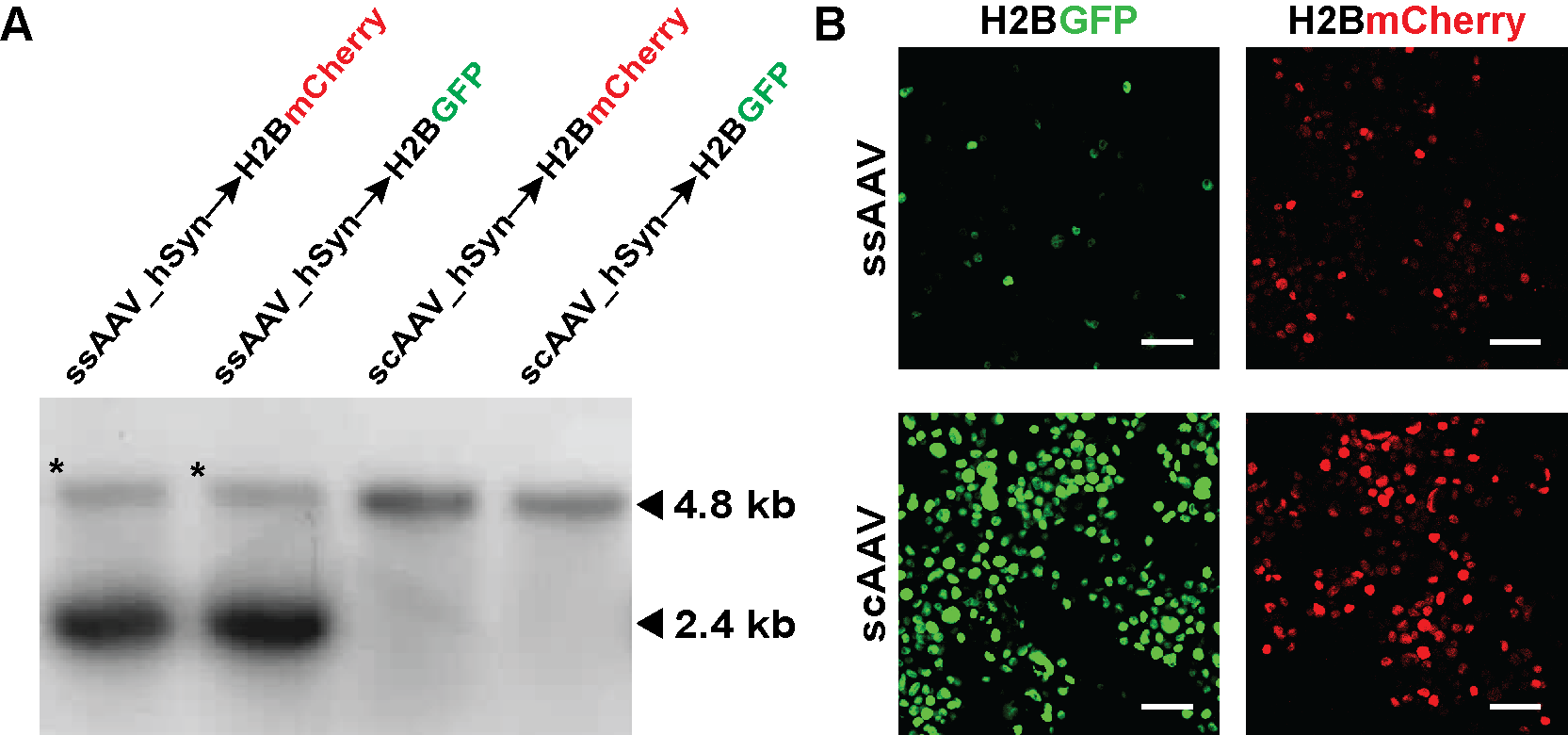

Supplement: Figure S3 — In vitro characterization of single stranded and self-complementary AAV8-hSyn-mCherry/GFP virus stocks. A) Viral vector DNA was isolated from purified AAV8 virus stocks and subsequently loaded on a denaturing alkaline agarose gel for size analysis. For single stranded vector genomes (ssAAV8) expected band sizes of 2422 bp (mCherry) and 2431 bp (GFP) were confirmed, whereas a doubling in band size to approximately 4.8 kb was observed for the respective self-complementary vector genomes (scAAV8) as expected. In samples from ssAAV8 virus stocks additional bands were detected at 4.8 kb as indicated by asterisks (*). These bands most likely originate from a subfraction of ssAAV8 particles containing dimeric genomes due to imperfect terminal resolution at the wtITR, a phenomenon frequently observed for packaging of small vector genomes with sizes below 2.5 kb. B) Neuro2A cells were transduced with single stranded and self-complementary AAV8-hSyn-mCherry/GFP virus stocks at an MOI of 2,5x10^5^ vector genomes per cell. 72 hours post transduction expression of fluorescent reporter genes was assessed by fluorescence microscopy, demonstrating superiority of scAAV8 virus stocks compared to ssAAV8 virus stocks as indicated by increasing numbers in GFP/mCherry-positive cells and enhanced transgene expression levels. Scale bars: 50 µm. (TIF) [file pone.0076310.s003.tif]

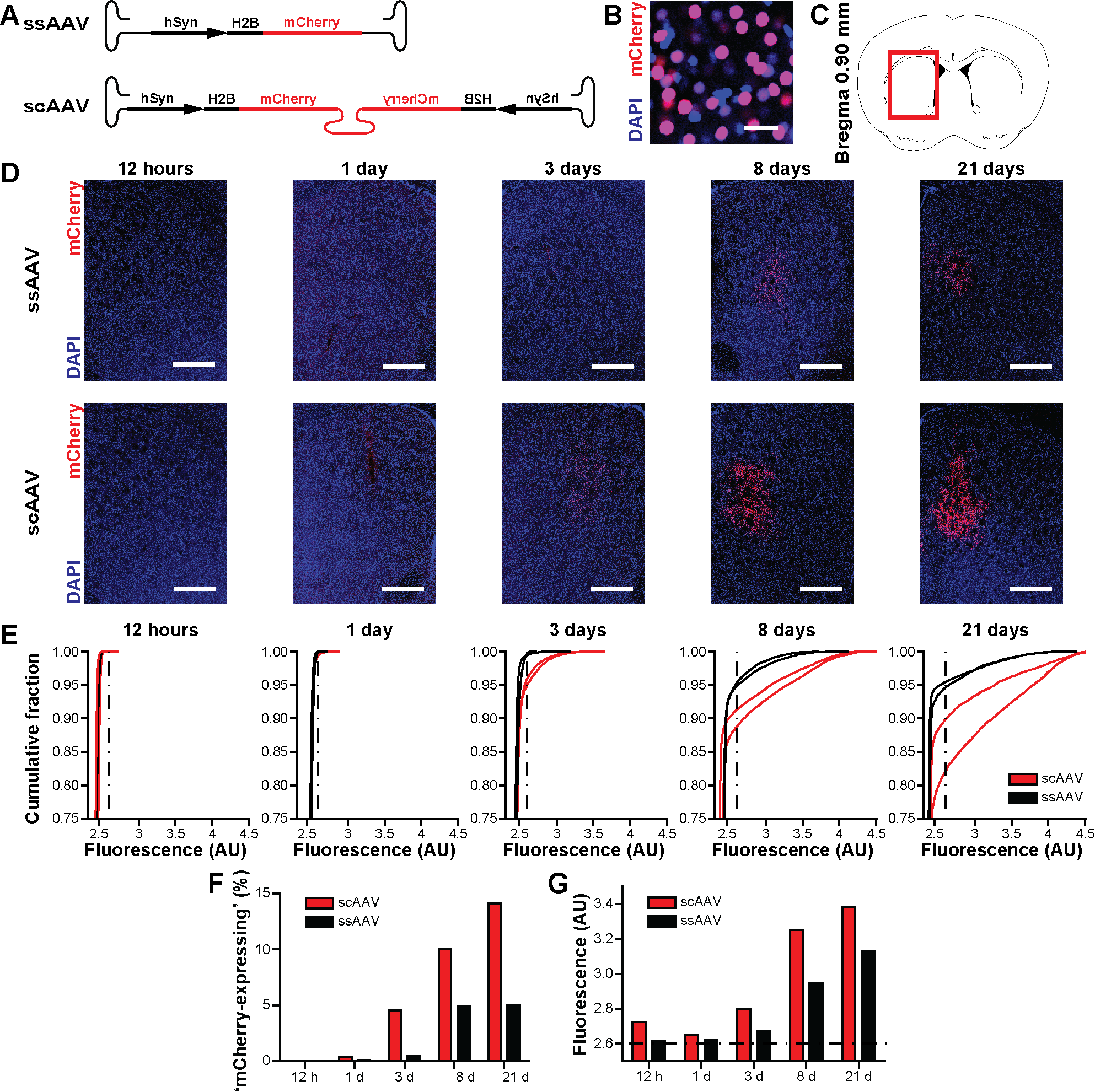

Supplement: Figure S4 — Time course of expression in striatal neurons transduced in vivo with ssAAV or scAAV coding for a red fluorescent protein. The experiment and analysis is analogous to the one shown in Figure 7, the only difference is that H2B was fused to mCherry instead of GFP. A) Schematics of the ssAAV and scAAV constructs driving expression of an H2B-mCherry fusion protein under the control of the neuronal human Synapsin 1 promoter that have been packaged in AAV8 capsids. B) High-magnification confocal fluorescence image of a coronal striatal section containing scAAV-transduced neurons that was stained with nuclear stain DAPI (blue) showing localization of the H2B-mCherry fusion protein (red) in the nucleus. Scale bar: 25 µm. C) Schematic of a coronal brain section in reference to Bregma with a red box indicating the areas shown in D. D) Confocal fluorescence images of coronal striatal sections stained with DAPI (blue) prepared at increasing time points following injection of ssAAV (top row) or scAAV (bottom row). Red mCherry expression in neurons becomes visible in ssAAV at eight days, whereas scAAV leads to earlier expression already at three days after injection. Scale bars: 250 µm. E) Cumulative distributions of mean fluorescence in the red channel of individual nuclei identified based on DAPI signal. Each line corresponds to the distribution of a brain section from one injected mouse (region of interest with a fixed size corresponding to approx. 15028±341 (mean±SEM) cells). Fluorescence measurements below 2.6 AU correspond mostly to non-expressing cells (e.g. 12 hour time point), whereas an appreciable fraction of cells in the striatum show intensities well above 2.6 AU as can be seen following longer incubation periods (e.g. 21 day time point). Dashed vertical line corresponds to 2.6 AU threshold. Two sections from two injected mice per virus type were analyzed on each time point. F) Fraction of cells within region of interest that show fluorescence levels higher than 2.6 [file pone.0076310.s004.tif]
